# Exploring the role of m ^6^ A writer RBM15 in cancer: a systematic review

**DOI:** 10.3389/fonc.2024.1375942

**Published:** 2024-06-10

**Authors:** Yuan Cao, Guanzhen Qiu, Yu Dong, Wei Zhao, Yong Wang

**Affiliations:** ^1^ Fourth Department of Orthopedic Surgery, Central Hospital Affiliated to Shenyang Medical College, Shenyang, Liaoning, China; ^2^ Shenyang 242 Hospital, Shenyang, Liaoning, China

**Keywords:** RBM15, methyltransferase, m 6 A, cancer, regulatory mechanisms

## Abstract

In the contemporary epoch, cancer stands as the predominant cause of premature global mortality, necessitating a focused exploration of molecular markers and advanced therapeutic strategies. N6-methyladenosine (m^6^A), the most prevalent mRNA modification, undergoes dynamic regulation by enzymes referred to as methyltransferases (writers), demethylases (erasers), and effective proteins (readers). Despite lacking methylation activity, RNA-binding motif protein 15 (RBM15), a member of the m^6^A writer family, assumes a crucial role in recruiting the methyltransferase complex (MTC) and binding to mRNA. Although the impact of m^6^A modifications on cancer has garnered widespread attention, RBM15 has been relatively overlooked. This review briefly outlines the structure and operational mechanism, and delineates the unique role of RBM15 in various cancers, shedding light on its molecular basis and providing a groundwork for potential tumor-targeted therapies.

## 1 Introduction

Cancer is the primary cause of premature mortality worldwide in the 21st century, posing a substantial barrier to improving human life expectancy (1). Calculations and data analysis from the Global Cancer Observatory (GCO) database underscore the substantial burden cancer will impose on both low- and middle-income nations and the global population over the next five decades (2). Therefore, the prioritization of efficient molecular markers to elucidate tumorigenic and progression mechanisms, along with the exploration of advanced early diagnostic and therapeutic approaches, is of utmost importance (3). Recently, m^6^A modification has emerged as a key player in cancer development, propelling ongoing epigenetic investigations into its association with cancer (4–6).

More than 150 chemically distinct RNA modifications have been recognized (7). m^6^A, first identified in the 1970s in eukaryotic messenger RNAs (mRNAs) and viral nuclear RNAs, is one of the most widespread and evolutionarily conserved internal cotranscriptional modifications in eukaryotic RNAs (8–11). Operating at the post-transcriptional level, this modification is dynamically regulated by enzymes referred to as writers, erasers, and readers (8, 12–14). The MTC consists of proteins known as “writers”, including methyltransferase-like 3 (METTL3), METTL14, Wilms’ tumor 1-associating protein (WTAP), RBM15, vir-like m^6^A methyltransferase associated (VIRMA; also known as KIAA1429), METTL16, zinc finger CCCH-type containing protein 13 (Zc3h13), and Hakai (15–21). Particularly noteworthy is the ability of METTL3 and METTL14 to form a stable heterodimer in a 1:1 ratio, underscoring their pivotal roles within the MTC (22). WTAP facilitates the recruitment of the METTL3-METTL14 heterodimer to specific target mRNAs (22–24). Recent studies have extensively reported that MTCs, including RBM15, play a crucial role in the development of various cancers (25–27).

RBM15, also recognized as OTT or OTT1 is a member of the split-end (SPEN) family of proteins involved in cell fate determination (28). Initially identified as an ectopic gene in pediatric acute megakaryocytic leukemia (29, 30), RBM15, despite lacking methylation activity, plays a critical role in recruiting MTCs and facilitating their binding to target mRNAs as a member of the m^6^A writers (31). Beyond its implications in hematopoiesis and cardio-splenic development in mice (32, 33), RBM15 is implicated in various biological functions, including alternative splicing, nuclear export, and X chromosome inactivation (19, 34, 35). Recent studies have revealed the involvement of RBM15 in cellular biological behaviors such as proliferation, invasion, migration, and apoptosis in various cancers, like acute megakaryocytic leukemia (36), colorectal cancer (37), ovarian cancer (38), laryngeal squamous cell carcinoma (25) and osteosarcoma (39). High expression levels of RBM15 are typically correlated with a poor prognosis in patients with malignancies (40). In this review, we will focus on the individual function of RBM15 and its related mechanisms, highlighting the current status of RBM15 regulatory mechanisms in tumors and providing researchers with new ideas for tumor therapy.

## 2 Structural features of the RBM15

**Figure 1:**
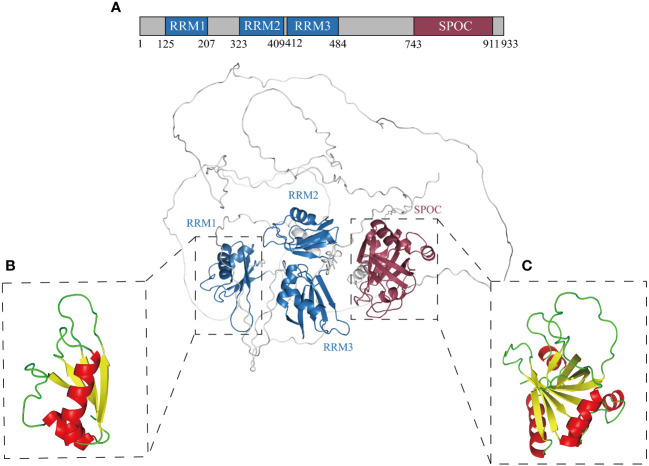
Structural model of RBM15 comprising three RRMs and one SPOC domain. **(A)** Schematic representation of RBM15 protein domains: RRM1 (aa 125 ~ 207), RRM2 (aa 323 ~ 409), RRM3 (aa 412 ~ 484), Spen orthologue and paralogue C-terminal (SPOC, aa 743 ~ 911). **(B)** Protein structural model of RBM15 RRM1: composed of 2 α-helices (red) and 4 β-strands (yellow). **(C)** Protein structure model of RBM15 SPOC: composed of 4 α-helices (red) and 7 β-strands (yellow). All structures were generated and colored using PyMOL version 2.5.4 (www.pymol.org).

RBM15, an RNA-binding protein, is situated within the 1p13.2 region of the human chromosome, characterized by 19 exons and 18 introns (41). RBM15 exhibits a typical SPEN family protein-like structure, comprising three highly conserved N-terminal RNA recognition motifs (RRM) and a spen orthologue and paralogue C-terminal (SPOC) domain (42, 43) (**Figure 1A**). The RRM folding structure includes four antiparallel β-strands and two α-helices (44) (**Figure 1B**). The RRM domain is highly prevalent in eukaryotes and plays an essential role in post-transcriptional splicing, translation, nuclear export, and mRNA stabilization (45, 46). The SPOC domain, characterized by seven β-strands and four α-helices (**Figure 1C**), has been demonstrated to influence several facets of mammalian gene expression, encompassing transcription, RNA modification, RNA export, and X-chromosome inactivation (47, 48). Recent discovery revealed that the SPOC domain serves as a phosphoserine binding module, with conserved motifs on its surface specifically recognizing the C-terminal domain (CTD) phosphorylation tag of RNA polymerase II (RNA Pol II) (42). RBM15 SPOC domain was shown to predominantly regulate m^6^A modification and enhance mRNA stability by binding to the m^6^A reader (48). Furthermore, the SPOC domain engages with a range of factors, including histone lysine methyltransferase SETD1B, nuclear RNA export factor 1 (NXF1), and DEAD-box protein 5 (DBP5), thereby participating in RNA transcription, export, and RNA-related metabolism (35, 49, 50). In conclusion, the RRM domain and the SPOC domain of RBM15 can mediate a variety of protein interactions and play a key role as a bridge in the regulation of gene expression.

## 3 Mechanisms underlying the function of RBM15

### 3.1 RBM15 serves as a writer in m^6^A

**Figure 2:**
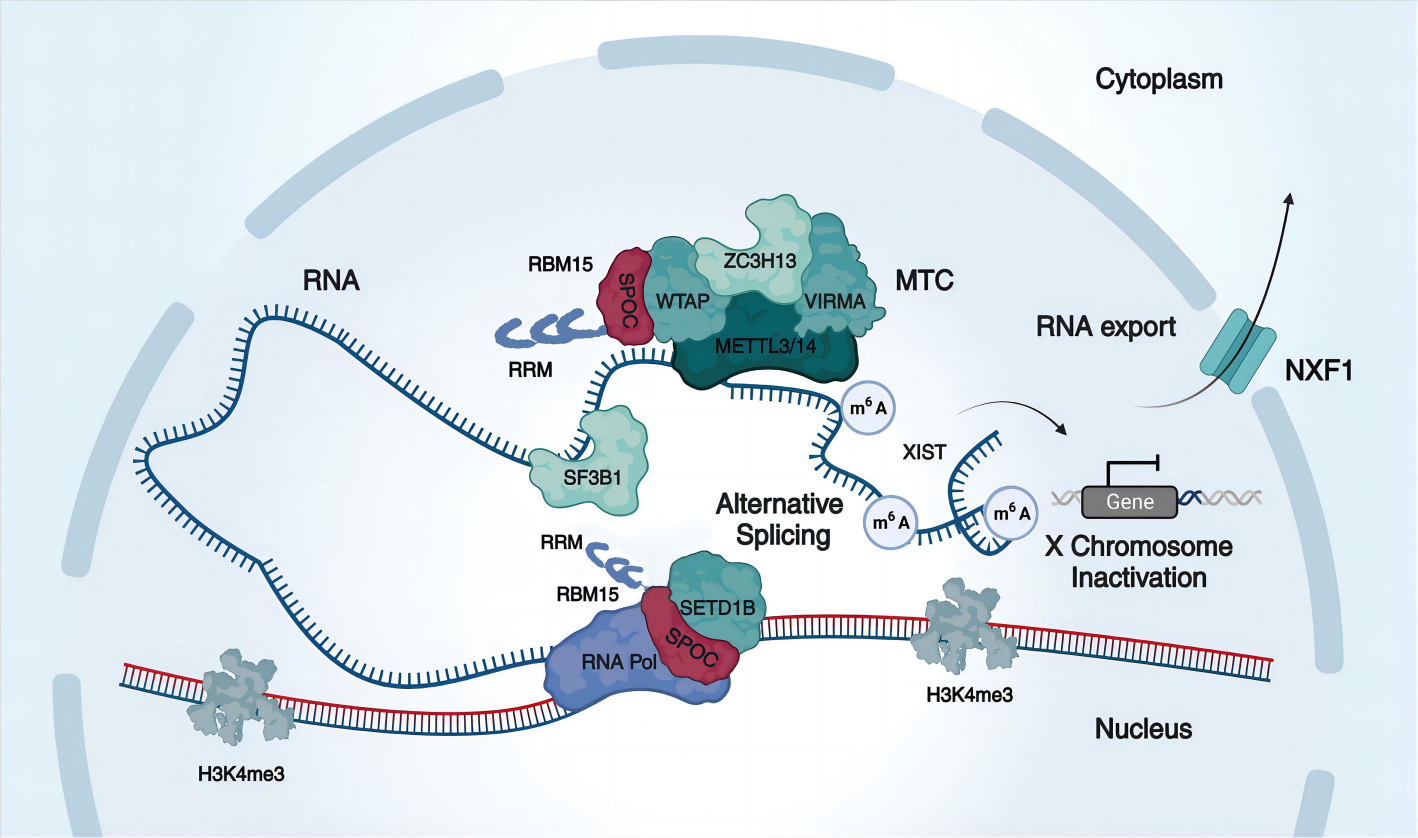
Mechanisms of RBM15 in m^6^A modification, alternative splicing, and RNA export. RBM15 comprises three RRMs and a SPOC domain. It collaborates with other m^6^A writers, including METTL3, METTL14, WTAP, VIRMA, and ZC3H13, to form a methyltransferase complex (MTC) that promotes methylation. For example, RBM15 promotes X-chromosome inactivation by enhancing XIST methylation levels. Additionally, RBM15 recruits the splicing factor SF3B1 to participate in alternative splicing, and RBM15 can target the histone H3K4me3 methyltransferase SETD1B to RNA via the SPOC domain, thereby regulating selective splicing via histone modifying enzymes. Furthermore, RBM15 promotes the nuclear export of mRNA by binding to the nuclear export factor NXF1.

In the context previously discussed, RBM15 assumes a pivotal role in the constitution of the MTC, guiding the METTL3/METTL14 complex to specific mRNA target sites (51) (**Figure 2**). Furthermore, RBM15 exhibits a selective binding affinity to U-rich sequences on mRNAs, directing them to distinct localization sites and thereby promoting m^6^A adenosine ribonucleotide methylation consensus motifs (19, 51).

Moreover, WTAP emerges as a robust candidate for interaction with RBM15, serving as an essential link in MTC recruitment by RBM15 (52). The interaction dynamics between the METTL3-METTL14 complex and RBM15 are intricately dependent on WTAP levels (19). It is noteworthy that the depletion of WTAP significantly diminishes or interrupts the binding interaction between RBM15 and the METTL3-METTL14 complex (19, 53–55). Subsequent experiments have elucidated that WTAP harbors a phosphorylated LSETD motif, emphasizing the potential dependence of the critical link between the RBM15 SPOC domain and WTAP on this phosphorylated motif (42).

RBM15, acting as a crucial mediator of m^6^A modifications, plays a multifaceted role in various biological processes. It collaborates with its analog RBM15b to recruit WTAP and METTL3-METTL14 to the long non-coding RNA X-inactive specific transcript (XIST) m^6^A regions, elevating XIST methylation levels and facilitating XIST-mediated gene silencing (19, 47, 56–58) (**Figure 2**). Interestingly, in cases of reduced RBM15 expression, RBM15b demonstrates functional compensation due to substantial sequence and structural domain similarity (19). Noteworthy XIST methylation reduction is observed only when both RBM15 and RBM15b are concurrently knocked down (19). Moreover, RBM15 orchestrates the m^6^A-mediated regulation of BAF155, contributing to the normal development of the mammalian cerebral cortex (54). RBM15 also regulates the expression of CLDN4 in mice, exerting influence on insulin sensitivity and promoting insulin resistance in gestational diabetic mice (59).

The intricate interplay between RBM15 and m^6^A readers plays a crucial role in orchestrating the intricate m^6^A regulatory mechanism. For instance, RBM15 can interact with IGF2BP1 to facilitate the post-transcriptional activation of YES proto-oncogene 1 (YES1), thus contributing to the regulation of hepatocellular carcinoma progression (60). Additionally, RBM15 may interact with Insulin-like growth factor 2 mRNA-binding protein 3 (IGF2BP3) and collaborate in the m^6^A modification of TMBIM6, consequently promoting the malignant progression of laryngeal squamous cell carcinoma (25). In conclusion, RBM15 is involved in the composition of MTCs in a WTAP-dependent manner and recruits MTCs to specific sites to promote m^6^A methylation, which plays a key regulatory role in a variety of biological functions.

### 3.2 RBM15 controls alternative splicing

Alternative splicing, facilitated by spliceosome complexes binding to RNA Pol II transcripts, constitutes a pivotal factor contributing to the intricate complexity of the transcriptome in multicellular eukaryotes (61, 62). Within nuclear speckles, recognized as crucial depots for numerous splicing factors, both RBM15 and RBM15b are situated (52). RBM15 exhibits its influence by binding to specific intronic sites in pre-mRNA and modulating alternative splicing through the recruitment of splicing factors (34) (**Figure 2**). A notable instance involves RBM15 orchestrating alternative splicing by enlisting the splicing factor SF3B1 to a distinct splice site in the c-Mpl intron, leading to the upregulation of the c-Mpl truncation isoform upon RBM15 knockdown (34). Additionally, RBM15 may govern alternative splicing via chromatin modifications. Its interactions with Hdac3 and the histone methyltransferase SETD1B exemplify this (63), influencing c-Mpl RNA and chromatin interactions, thereby regulating H4 acetylation and H3K4me3 marks (64) (**Figure 2**). Noteworthy is the observation that inhibiting histone deacetylase or histone methyltransferase levels significantly heightens the abundance of truncated isoforms of c-Mpl (64, 65). Upstream in the regulatory cascade, protein arginine methyltransferase 1 (PRMT1) methylates RBM15, instigating its ubiquitination and subsequent degradation by subunit 4 of the CCR4-NOT transcriptional complex (CNOT4) (34). This intricate process potentially serves as a pathogenetic mechanism in hematopoietic malignancies.

Alternative splicing orchestrated by RBM15 is essential for megakaryocyte differentiation. For instance, the depletion of RBM15 disrupts the selective splicing of GATA1, leading to the generation of truncated GATA1 isoforms (34). These truncated isoforms hinder the differentiation of progenitors into mature megakaryocytes, a crucial process in the pathogenesis of leukemia (34). Additionally, the transcription factor TAL1 holds a critical role in the differentiation of megakaryocyte-erythroid progenitors. The strong interaction between the SF3B1^K700E^ mutant and RBM15 can result in the dysregulation of alternative RNA splicing of TAL1, ultimately impeding erythropoiesis (66). In summary, RBM15 regulates alternative splicing by recruiting splicing factors and interacting with histone-modifying enzymes, thereby governing the splicing process and participating in diverse functions (34, 64).

### 3.3 RBM15 promotes the nuclear export of mRNA

RBM15, a member of the SPEN family, possesses a distinctive SPOC domain responsible for governing nuclear export through interactions with diverse proteins (28) (**Figure 2**). For example, EB2 interacts with RBM15/b and the SPEN SPOC domain, thus facilitating the nuclear export of viral mRNA (28). The RNA transport element (RTE) enhances RNA binding to the mRNA export receptor NXF1, with this process mediated by the interaction with RBM15 (35). Acting as a bridge, RBM15 connects RTE-containing RNA to NXF1, consequently amplifying the nuclear export of RNA (35). Moreover, the DBP5 plays a crucial role in providing the basic direction of nuclear export by specifically recognizing NXF1 through RBM15, allowing NXF1 to traverse the nuclear pore complex and enter the cytoplasm (35, 49). In summary, RBM15 actively engages in and facilitates cellular nuclear export. Nevertheless, when RBM15/b is subjected to knockdown, the nuclear export of mRNA appears to persist, raising questions about whether RBM15 solely aids export factors in enhancing the stability of their interactions (35, 49).

## 4 Role of RBM15 in cancer

**Figure 3:**
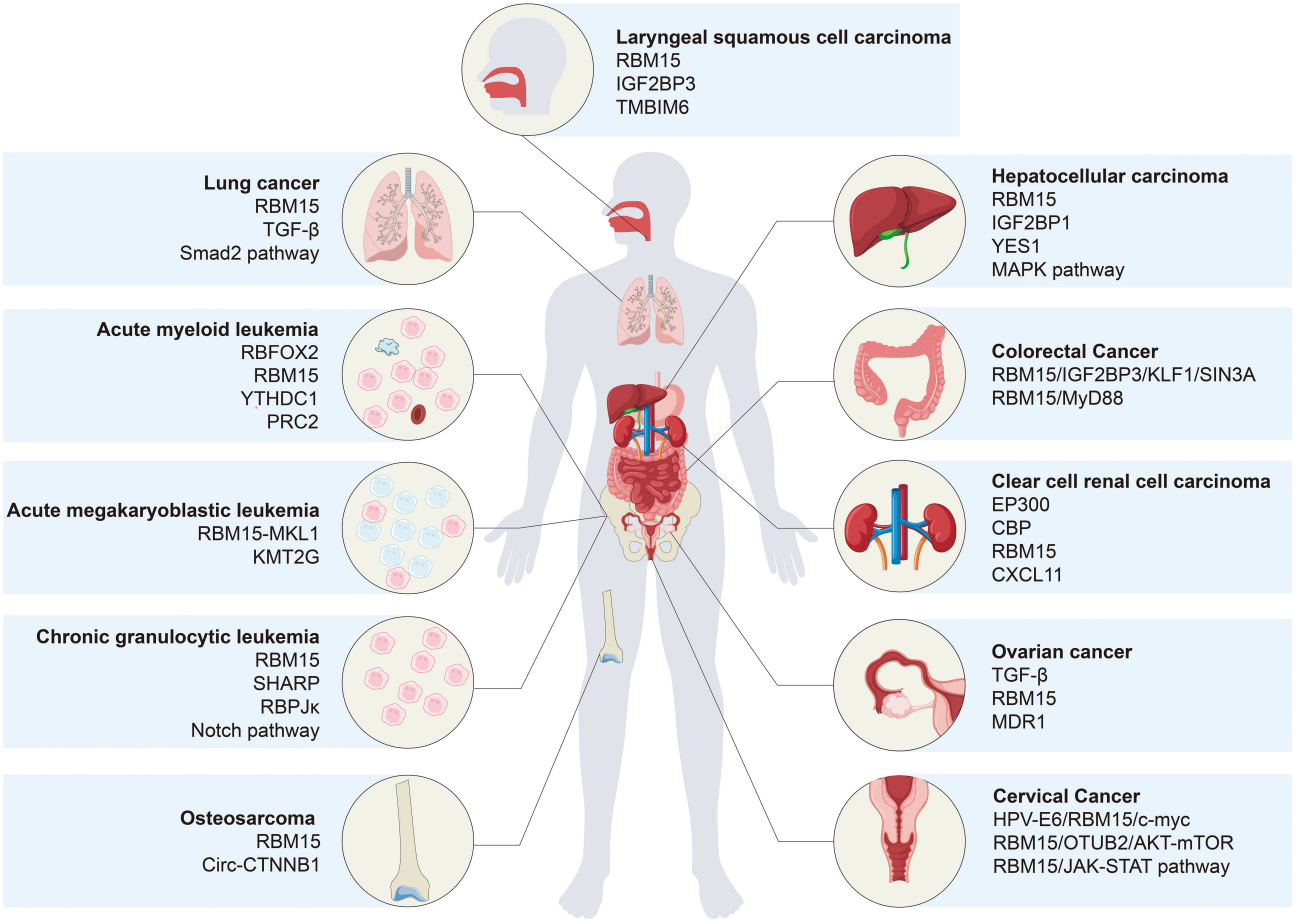
Molecular regulatory mechanisms of RBM15 in various types of cancer. In addition to conventional targeted regulation, RBM15 collaborates with m^6^A readers, such as IGF2BP1, IGF2BP3, and YTHDC1, to govern the malignant progression of cancer. Furthermore, RBM15 directs cancer progression by modulating various pathways, including TGF-β/Smad2, Notch, MAPK, and AKT/mTOR pathways.

Recent evidence underscores the pivotal role of RBM15 in cancer, where it predominantly functions as a methyltransferase, enhancing the stability of target mRNAs through m^6^A modification, thereby contributing to the initiation and progression of diverse cancers. In addition, RBM15 also regulates cancer through signaling pathways or other modifications. In this review, we present a succinct summary of recent discoveries elucidating the expression of RBM15 in different tumors and its corresponding molecular regulatory mechanisms (**Table 1** and **Figure 3**).

**Table 1:** Molecular regulatory mechanisms of RBM15 in cancer.

Cancer type	Regulator	Targets	Molecular mechanism	Cellular function	Ref.
Acute myeloid leukemia	RBFOX2	PRC2	RNA stabilization by YTHDC1	Proliferation, survival, and promotion of myeloid differentiation	(67)
Acute megakaryoblastic leukemia		KMT2G	Enhanced pathogenic activity of RBM15-MKL1	Proliferation and survival	(65)
Chronic granulocytic leukemia		RBPJκ	Activation of the Notch pathway	Proliferation	(68)
Kaposi’s sarcoma		ORF57, ORF59	increase the stability and promote nuclear export	Viral proliferation	(69)
Hepatocellular carcinoma		YES1	Activation of the MAPK pathway	Proliferation and migration	(60)
Colorectal cancer		MyD88		Proliferation and invasion	(37)
Colorectal cancer		KLF1	Activation of transcription.	Proliferation, invasion, and migration	(70)
Pancreatic adenocarcinoma				Proliferation, invasion, and migration	(71)
Cervical cancer	HPV E6	c-myc	Inhibition of RBM15 autophagy	Proliferation	(72)
Cervical cancer		Otubain 2	Activation of the AKT/mTOR pathway	Proliferation, invasion, apoptosis, and migration	(73)
Cervical cancer			Activation of the JAK-STAT pathway	proliferation, invasion, and migration	(74)
Ovarian cancer	TGF-β/Smad2	MDR1	Downregulation of RBM15 expression	Re-sensitization of PTX-resistant cells	(75)
Clear cell renal cell carcinoma	EP300/CBP	CXCL11	Macrophage infiltration and M2 polarization	Proliferation, invasion, migration, and epithelial mesenchymal transition	(76)
Lung cancer		TGF-β/Smad2	Regulation of ferroptosis	Proliferation, invasion, and migration	(77)
Laryngeal squamous cell carcinoma		TMBIM6	RNA stabilization by IGF2BP3	Proliferation, invasion, migration, and apoptosis	(25)
Osteosarcoma	CTNNB1		Promotion of aerobic glycolysis	Tumor growth, invasion, and metastasis	(39)

### 4.1 Acute myeloid leukemia

Acute myeloid leukemia (AML) stands out as the preeminent malignancy affecting hematopoietic stem cells, marked by a notably unfavorable prognosis (78). Recent research has highlighted a substantial correlation between RBM15 expression levels and survival in AML patients, associating elevated RBM15 expression with shorter survival (67). Notably, a critical aspect of RBM15 function was uncovered in that RBM15 was recruited by RBFOX2 and established an interaction with the m^6^A reader YTH domain-containing proteins 1 (YTHDC1) (67). This interaction facilitated the recruitment of polycomb repressive complex 2 (PRC2) to the binding site of RBFOX2, leading to chromatin silencing and transcriptional repression (67). Of note, the expression levels of RBM15 and RBFOX2 were positively correlated in cancer patients. Furthermore, down-regulation of RBFOX2 significantly impedes the survival and proliferation of AML cells and induces myeloid differentiation (67).

### 4.2 Acute megakaryoblastic leukemia

Acute megakaryocytic leukemia (AMKL) is a subtype of acute myeloid leukemia primarily characterized by the presence of platelet-producing megakaryocytes within the bone marrow (79). This disease is prevalent in children and is associated with an unfavorable prognosis (79–81). The gene fusion product involving RBM15 and megakaryocytic leukemia 1 (MKL1), termed the RBM15-MKL1 fusion protein (also referred to as OTT-MAL), was initially identified in a pediatric patient with acute megakaryoblastic leukemia harboring the t(1;22)(p13;q13) translocation (82). The etiology of this malady is notably intricate, and investigations have revealed that the RBM15-MKL1 fusion protein interacts with Setd1b histone H3-Lys4 methyltransferase (also recognized as KMT2G). This interaction is contingent on the intact RBM15 SPOC domain and enhances its leukemic activity in megakaryocytes (65).

Furthermore, RBM15 plays multiple roles in the hematopoietic system and may also contribute to disease pathogenesis (83, 84). As an illustration, RBM15 interacts with SMRT/HDAC1-related inhibitory protein (SHARP) and is associated with the recombinant signal-binding protein RBP-Jκ. This interaction activates Notch-regulated gene expression, thereby inhibiting myeloid differentiation in hematopoietic cells (83). Additionally, RBM15 plays a role in hematopoietic stem cells (HSC) and contributes to megakaryocyte development by regulating the downstream target c-myc (85, 86). Furthermore, It has been shown that antisense RBM15 (AS-RBM15) finely modulates megakaryocyte differentiation by elevating the translation level of the RBM15 protein (87). Up-regulation of AS-RBM15 expression promotes terminal differentiation of megakaryocytes, while down-regulation has the opposite effect. Nevertheless, the connection of these findings to the pathogenesis of AMKL requires further investigation.

### 4.3 Chronic granulocytic leukemia

Chronic granulocytic leukemia (CML) is one of the most malignant diseases in the hematopoietic system, presenting a grave threat to patients (88). Related researchers revealed that the average expression level of RBM15 was notably higher in acute-phase CML cells when compared to those in the chronic and accelerated phases of the disease (68). Diminishing RBM15 levels demonstrated the capacity to impede the growth and proliferation of CML cells, impede the cell cycle, and induce apoptosis (68). Furthermore, evidence suggested that RBM15 might, in part or entirely, facilitate the malignant progression of CML through the Notch signaling pathway mediated by RBPJκ. This pathway is postulated to wield a crucial influence in the etiology of hematopoietic malignancies (68, 83).

### 4.4 Kaposi’s sarcoma

Kaposi’s sarcoma (KS) is a multicentric tumor arising from the endothelial cells of lymphatic vessels (89). The virulence genes ORF57 and ORF59 are vital contributors to the growth and proliferation of KS (90), and their expression levels are closely associated with RBM15 (69). Research has revealed that RBM15 plays a key role in enhancing the production of ORF57 nuclear transcripts. Inhibition of RBM15 hampers the production of ORF57 mRNA, resulting in a decrease in the overall RNA level of ORF57 (91). In addition, RBM15 and ORF57 interacted with the 5’ MRE of ORF59 to increase the stability of ORF59 mRNA and promote its nuclear export (90), preventing the overaccumulation of ORF59 mRNA in the nucleus and maintaining the balance between the nuclear and cytoplasmic levels of ORF59 mRNA (69). However, further experiments are needed to confirm that RBM15 regulates the expression of ORF57 and ORF59, thereby promoting the growth and proliferation of KS.

### 4.5 Hepatocellular carcinoma

Hepatocellular carcinoma (HCC) is the predominant primary liver malignancy, distinguished by elevated malignancy, morbidity, and mortality rates (92). RBM15 exhibits high expression levels in HCC, indicative of an unfavorable prognosis (60, 93). The depletion of RBM15 significantly impedes the growth of HCC cells (60). Notably, it uncovered a crucial aspect of RBM15 function, demonstrating its pivotal role in the post-transcriptional activation of the YES1 through interaction with the m^6^A reader IGF2BP1 (60). This intricate interplay subsequently activates the mitogen-activated protein kinase (MAPK) pathway, thereby fostering the progression of HCC (60).

### 4.6 Colorectal cancer

Colorectal cancer (CRC) is the third most common malignancy and the second most lethal cancer in the world, and its incidence is increasing at an alarming rate (94). Research indicates that the expression of RBM15 in CRC tissues is significantly higher than that in nearby non-tumor tissues, and elevated expression of RBM15 is closely associated with poor prognosis, while inhibition of RBM15 expression significantly suppresses the proliferation and invasion of CRC cells (70). Recently, more and more studies have focused on the mechanistic investigation of RBM15 in CRC. For example, RBM15 increased the methylation level of MyD88 through m^6^A modification, which promoted the proliferation and invasion of CRC cells (37). In addition, RBM15 regulates the expression and enhances the stability of KLF1 mRNA by interacting with the m^6^A reader IGF2BP3, which activates the transcription of the downstream target SIN3A and ultimately promotes the proliferation, invasion, and migration of CRC cells (70).

### 4.7 Pancreatic cancer

Pancreatic adenocarcinoma (PAAD) is a prominent and exceptionally malignant neoplasm of the digestive system, characterized by a 5-year survival rate of approximately 10% following diagnosis (95). RBM15 exhibited high expression across various pancreatic cancer cell lines, frequently coexisting with a propensity for T lymphocyte aggregation (41). Studies have shown that elevated RBM15 expression emerged as a significant contributor to unfavorable prognosis, and conversely, the suppression of the RBM15 demonstrates the potential to inhibit cancer cell proliferation, invasion, and metastasis to varying extents (41, 71). Intriguingly, elevated blood glucose levels may enhance RBM15 expression in pancreatic cancer. It is yet to be determined whether this phenomenon is associated with the increased energy demands of pancreatic cancer malignancy (41).

### 4.8 Cervical cancer

Cervical cancer is the fourth most prevalent gynecological cancer, and more than 99% of cases are attributed to human papillomavirus (HPV) (96). HPV-E6, one of the eight protein-coding genes associated with cervical carcinogenesis (97), has been shown to maintain high expression of RBM15 in cervical cancer cells by preventing its autophagic degradation (72). Recent studies have focused on the specific regulatory mechanisms of RBM15 in cervical cancer. For example, RBM15 interacts with the downstream target c-myc, enhancing its m^6^A modification, a process crucial in cervical cancer development (72). Intriguingly, the knockdown of HPV-E6 resulted in a reduction of c-myc mRNA expression and m^6^A modification levels in cervical cancer cells, which were subsequently reversed by the overexpression of RBM15 (72). In addition, RBM15-mediated m^6^A modification facilitated the expression of the oncogene Otubain 2 (OTUB2) in cervical cancer cells, which further activated AKT/mTOR signaling, thereby promoting the proliferation, migration, and invasion of cervical cancer cells (73). Furthermore, it has been hypothesized that RBM15 might play a role in promoting the proliferation, invasion, and migration of cervical cancer cells through its interaction with the JAK-STAT pathway. Nevertheless, additional validation is required (74).

### 4.9 Ovarian cancer

Ovarian cancer (OC) is the third most common gynecologic malignancy worldwide but accounts for the highest mortality rate among these cancers (98). Studies have shown that the expression of RBM15 is higher in OC tissues than in normal tissues, and that high expression of RBM15 is closely associated with the propensity to metastasize in OC (38, 75). Interestingly, RBM15 is overexpressed in paclitaxel (PTX)-resistant cells and depletion of RBM15 also restores the sensitivity of PTX-resistant cells (75). Activation of the TGF-β/Smad pathway was shown to interact with the RBM15 promoter and directly inhibit RBM15 expression in PTX-resistant ovarian cancer cells (75). Subsequent low expression of RBM15 resulted in a reduction in the m^6^A level of MDR1, a recognized major target for overcoming OC resistance (75, 99). In summary, RBM15, serving as a tissue biomarker for OC and PTX resistance, may open new therapeutic avenues for the treatment of PTX-resistant OC in the future.

Most evidence has unveiled the oncogenic role of RBM15 in OC, however, a different voice also indicated a contradictory view: ubiquitin-like modification activating enzyme 6 antisense RNA 1 (UBA6-AS1) can m^6^A-regulate UBA6 mRNA by enlisting the aid of RBM15, followed by the m^6^A reader IGF2BP1, which bolsters the stability of UBA6 mRNA (100). UBA6-AS1 curbed UBA6 self-degradation through m^6^A modification mediated by RBM15, thereby effectively impeding ovarian cancer proliferation, invasion, and metastasis (100). This suggests to us that RBM15 may be associated with a favorable prognosis in ovarian cancer. However, further experiments are warranted to validate this association. While controversy surrounds RBM15’s role in ovarian cancer, these findings suggest the potential for a future role wherein RBM15 acts as a tumor suppressor or a tumor suppressor cofactor.

### 4.10 Clear cell renal cell carcinoma

Clear cell renal cell carcinoma (ccRCC) is a prevalent adenocarcinoma originating from renal tubular epithelial cells, frequently associated with an unfavorable prognosis (68). Most investigations have revealed a heightened expression of RBM15 in both ccRCC cells and tissues, correlating with augmented proliferation, invasion, and metastasis of ccRCC cells (76, 101). Creb-binding protein (CBP) and EP300 are crucial transcriptional co-regulators implicated in cancer progression (102). It has been shown that they can facilitate the enrichment of the RBM15 promoter, inducing histone 3 acetylation modification and the subsequent upregulation of RBM15 expression. Furthermore, RBM15 plays a role in enhancing the stability of CXCL11 mRNA through m^6^A modification, consequently promoting macrophage recruitment and M2 polarization (76). Noteworthy is the observation that the knockdown of RBM15 led to a significant reduction in the m^6^A level of CXCL11 mRNA, thereby restraining the malignant behavior of ccRCC cells (76).

### 4.11 Lung cancer

Lung cancer stands out as the most prevalent global malignancy and the leading cause of cancer-related mortality among men (103), Among its subtypes, lung adenocarcinoma (LUAD) constitutes approximately half of the total incidence (104). LUAD cells exhibit significantly heightened levels of RBM15 expression, and this upregulation strongly correlates with diminished overall survival rates (105, 106). Notably, RBM15’s potential to promote the malignant behavior of lung cancer is linked to its antagonistic relationship with SETD2, a recognized favorable prognostic indicator for LUAD (107). A recent study has further proposed that the knockdown of RBM15 effectively reduces the levels of TGF-β and Smad2, and promotes ferroptosis by regulating genes related to the iron concentration process, thus inhibiting proliferation, migration, invasion, and tumor growth (77). The proposition of targeting RBM15 opens new avenues for future directions in lung cancer treatment.

### 4.12 Laryngeal squamous cell carcinoma

Laryngeal squamous cell carcinoma (LSCC), a highly malignant tumor of the respiratory tract, holds the unenviable position of being the second most common head and neck cancer with a particularly dismal prognosis (108). LSCC tissues conspicuously exhibit elevated levels of RBM15 expression, significantly associated with a poor prognosis (20). Interestingly, inhibition of RBM15 by knockdown significantly impedes the invasion and migration capabilities of LSCC cells (25). Recently, a remarkable discovery demonstrated that RBM15 plays a critical role in promoting the methylation process of TMBIM6, consequently facilitating the malignant progression of LSCC (25). In addition, the m^6^A reader IGF2BP3 recognizes the m^6^A tag and exerts its influence by fortifying the stability of TMBIM6 mRNA. Notably, when the expression of RBM15 and IGF2BP3 was knocked down, a significant decrease in the expression level of TMBIM6 mRNA was observed (25).

### 4.13 Osteosarcoma

Osteosarcoma is an exceedingly uncommon primary malignancy of the skeletal system, primarily afflicting adolescents between the ages of 10 and 25 years (109). In osteosarcoma cells, RBM15 exhibits elevated expression levels, a phenomenon notably linked to an unfavorable prognosis (110). Mechanistic studies on how RBM15 regulates osteosarcoma are scarce, but a recent study suggested that RBM15 directly interacts with Circ-CTNNB1, thereby increasing the level of m^6^A modification of genes associated with aerobic glycolysis, and ultimately facilitating the glycolytic process (39). This augmentation in aerobic glycolysis unequivocally provides a survival advantage to osteosarcoma cells (39). Yet, the precise regulatory mechanism underpinning this phenomenon remains to be comprehensively elucidated.

## 5 Conclusions and perspectives

Cancer is characterized by numerous hallmark behaviors such as uncontrolled proliferation, evasion of cell death, angiogenesis, invasion, metastasis, metabolic dysregulation, and immune evasion (111). The most prevalent RNA modification in eukaryotes is m^6^A, which can determine the fate of the modified RNA (112). Methyltransferases have garnered substantial research interest due to their ability to catalyze RNA modifications, their involvement in tumor initiation and progression, and their potential as therapeutic targets in cancer (12). For instance, METTL3 may govern colorectal cancer metastasis by modulating the METTL3/miR-1246/SPRED2 axis (113), Additionally, METTL14 regulates USP48, enhancing SIRT6 stability via m^6^A modification, thereby restraining the malignancy of HCC (114).

RBM15, belonging to the SPEN family and distinguished by its specific SPOC domain, assumes a key role in recruiting MTC to specific sites, thereby facilitating m^6^A methylation (42, 51). Additionally, RBM15 extends its impact beyond alternative mRNA splicing and nuclear transport, encompassing a spectrum of biological functions mediated by m^6^A methylation. These functions encompass Xist-mediated chromosome inactivation and the mediation of the degradation of the chromatin remodeling factor BAF155 (19, 34, 35, 54). In addition, RBM15 is an oncogene in most cancers, and down-regulation of RBM15 can effectively inhibit cancer progression (37, 39, 81). Based on emerging evidence of their role in cancer and molecular mechanisms, m^6^A regulators have attracted increasing attention from researchers as therapeutic targets.

Certainly, RBM15 also assumes a significant role in non-neoplastic diseases. For instance, RBM15 can accelerate the progression of diabetic nephropathy by modulating cell proliferation, inflammation, and oxidative stress through activation of the AGE-RAGE pathway (82). In addition, RBM15 triggers abnormal immune responses and lymphopenia, thereby exacerbating inflammatory reactions in COVID-19 through the regulation of multiple downstream target genes (83). Consequently, RBM15 holds promise as a prospective target for the treatment of malignancies and a wide range of diseases. Manipulation of RBM15 levels, whether through direct or indirect means, is expected to improve patient prognosis in the future.

In this article, we provide a comprehensive review of RBM15 expression in various cancer types, exploring its impact on prognosis and the underlying molecular mechanisms. However, RBM15-targeted therapies are still in their infancy and there are still significant gaps in the understanding of its upstream regulation and downstream targeting. It remains unclear whether it occurs through the m^6^A pathway in various tumor models, which is crucial for clinical translational applications as well as the development of disease therapies. It should be emphasized that although RBM15 has a specific SPOC domain, the relevance of its structure to disease and the specific upstream and downstream regulatory mechanisms are still rarely mentioned. In the future, we can pay more attention to how the SPOC domains are removed or lose their functions, understand how the SPOC domain regulates the synthesis and fate of mRNAs at the molecular level, and potentially discover potential targets for new therapeutic approaches. In addition, RBM15b is an analog of RBM15, which together with RBM15 plays a key recruiting role in the process of m^6^A methylation. However, the synergistic effects of RBM15b on RBM15 in disease regulation, as well as their potential to jointly induce cancer and promote tumor cell growth, have rarely been analyzed in detail. In the future, we may need additional transgenic mouse models to test the specific roles of these two analogs and their synergistic effects *in vivo*.

In conclusion, RBM15 is involved in a wide range of biological processes and its importance in cancer regulation is increasing. Therefore, it is imperative to elucidate the complex roles of RBM15 in cancer and to exploit its potential in targeted tumor therapy to bridge the gap between research findings and clinical translation, ultimately improving the prognosis of cancer patients.

## Author contributions

YC: Conceptualization, Investigation, Software, Visualization, Writing – original draft, Writing – review & editing. GQ: Supervision, Validation, Writing – review & editing. YD: Supervision, Validation, Writing – review & editing. WZ: Supervision, Validation, Writing – review & editing. YW: Conceptualization, Investigation, Resources, Supervision, Validation, Visualization, Writing – review & editing.
